# Functional reorganization of monoamine transport systems during villous trophoblast differentiation: evidence of distinct differences between primary human trophoblasts and BeWo cells

**DOI:** 10.1186/s12958-022-00981-8

**Published:** 2022-08-04

**Authors:** Veronika Vachalova, Rona Karahoda, Martina Ottaviani, Kasin Yadunandam Anandam, Cilia Abad, Christiane Albrecht, Frantisek Staud

**Affiliations:** 1 Department of Pharmacology and Toxicology, Faculty of Pharmacy in Hradec Kralove, Charles University, Hradec Kralove, Czech Republic; 2 Institute of Biochemistry and Molecular Medicine, University of Bern, Bern, Switzerland; 3 Swiss National Centre of Competence in Research, NCCR TransCure, University of Bern, Bern, Switzerland

**Keywords:** Trophoblast, Placenta, Monoamines, Neuroplacentology, Membrane transport, Cell differentiation

## Abstract

**Background:**

Three primary monoamines—serotonin, norepinephrine, and dopamine—play major roles in the placenta-fetal brain axis. Analogously to the brain, the placenta has transport mechanisms that actively take up these monoamines into trophoblast cells. These transporters are known to play important roles in the differentiated syncytiotrophoblast layer, but their status and activities in the undifferentiated, progenitor cytotrophoblast cells are not well understood. Thus, we have explored the cellular handling and regulation of monoamine transporters during the phenotypic transitioning of cytotrophoblasts along the villous pathway.

**Methods:**

Experiments were conducted with two cellular models of syncytium development: primary trophoblast cells isolated from the human term placenta (PHT), and the choriocarcinoma-derived BeWo cell line. The gene and protein expression of membrane transporters for serotonin (SERT), norepinephrine (NET), dopamine (DAT), and organic cation transporter 3 (OCT3) was determined by quantitative PCR and Western blot analysis, respectively. Subsequently, the effect of trophoblast differentiation on transporter activity was analyzed by monoamine uptake into cells.

**Results:**

We present multiple lines of evidence of changes in the transcriptional and functional regulation of monoamine transporters associated with trophoblast differentiation. These include enhancement of SERT and DAT gene and protein expression in BeWo cells. On the other hand, in PHT cells we report negative modulation of SERT, NET, and OCT3 protein expression. We show that OCT3 is the dominant monoamine transporter in PHT cells, and its main functional impact is on serotonin uptake, while passive transport strongly contributes to norepinephrine and dopamine uptake. Further, we show that a wide range of selective serotonin reuptake inhibitors affect serotonin cellular accumulation, at pharmacologically relevant drug concentrations, via their action on both OCT3 and SERT. Finally, we demonstrate that BeWo cells do not well reflect the molecular mechanisms and properties of healthy human trophoblast cells.

**Conclusions:**

Collectively, our findings provide insights into the regulation of monoamine transport during trophoblast differentiation and present important considerations regarding appropriate in vitro models for studying monoamine regulation in the placenta.

**Supplementary Information:**

The online version contains supplementary material available at 10.1186/s12958-022-00981-8.

## Background

Neuroplacentology is an emerging field of research focused on the placenta’s role in fetal brain development [1]. Growing evidence suggests that many neurobehavioral conditions originate during in utero development. Three primary monoamines conveyed by the placenta to the developing brain (serotonin, norepinephrine, and dopamine) play major roles as neurotransmitters in the placenta-brain axis [2–5]. In the prenatal period, these monoamines play significant roles in development of the fetal brain, and both cardiovascular and respiratory systems [6, 7]. In the placenta, monoamines regulate hormone synthesis and affect placental metabolism [8, 9]. They also mediate maternal adaptation by influencing the maternal neuroendocrine system [10]. Thus, proper monoamine levels in the fetoplacental unit are required to ensure precise fetal development and programming.

Analogously to the brain, the placenta is well equipped with transporters and enzymes that play major roles in maintenance of monoamine balances throughout gestation. These transporters are centralized in a layer of terminally differentiated cells, the multinucleated syncytiotrophoblast (STB), and polarized in the maternal-facing microvillous membrane (MVM) and fetal-facing basal membrane (BM) [11]. Most current knowledge on monoamine transporter expression in the placenta has been obtained from ex vivo uptake studies with isolated placental membranes, where functional expression of the high-affinity/low-capacity serotonin and norepinephrine transporters (SERT and NET, respectively) is confined to MVM [12–14]. Placental dopamine uptake is reportedly mediated by SERT or NET, as no reports either confirm or refute the presence of any dopamine transporter (DAT) in the placenta [15]. In addition, we have recently found that organic cation transporter 3 (OCT3), a low-affinity/high-capacity transporter localized in the BM, mediates serotonin uptake from the fetal circulation [16]. Studies from other tissues have suggested that norepinephrine and dopamine are substrates of OCT3 [17, 18], but this has not been confirmed in the placenta.

Beneath the STB lie the highly proliferative cytotrophoblasts (CTBs), which continuously renew and repair the epithelial layer. Processes involved in STB formation include asymmetrical cell division, biochemical differentiation, and fusion of CTBs, tightly guided by transcription factors, fusogenic proteins, cell adhesion molecules, extracellular matrix components, and soluble factors [19]. Several reports have described differences in transcriptomic profiles [20–23] and endocrine function [24, 25] between the two cell stages. A recent study shows that during this process substantial functional reorganization of villous trophoblast occurs, including changes in transcription patterns of genes encoding monoamine transporters [26]. As serotonin, norepinephrine, and dopamine have been shown to modulate cellular functions such as division and differentiation [27–29], we hypothesized that regulatory interplay occurs between trophoblast differentiation and placental monoamine transport systems.

Thus, in this study, we sought to characterize the expression and functional activity of monoamine transporters in placental cells and assess changes in cellular levels of monoamines associated with trophoblast differentiation. While placental membrane vesicles are valuable models to investigate some characteristics of transport systems, they have limited scope for addressing regulatory aspects affecting transporter functions. Highly purified primary human trophoblast (PHT) cells isolated from human term placenta are known to spontaneously undergo differentiation and fusion in culture, thus mimicking the differentiation in vivo. However, the choriocarcinoma-derived BeWo cell line is the most commonly used model for this purpose as it responds to cell fusion signals both morphologically and biochemically [30]. Therefore, using these two models, we aimed to elucidate the cellular complexity of monoamine transport in the human placenta. Collectively, our findings provide insights into regulatory aspects of changes in monoamine regulation associated with trophoblast differentiation, which potentially reflect their involvement in cellular functions in the placenta. They also provide important indications regarding appropriate in vitro models for studying monoamine regulation in the placenta.

## Methods

### Chemicals and reagents

^3^H-serotonin (80 Ci/mmol), ^3^H-norepinephrine (20 Ci/mmol), and ^3^H-dopamine (60 Ci/mmol) were purchased from M.G.P. (Zlín, Czech Republic). Forskolin was obtained from Scintila, s.r.o. (Jihlava, CZ). Paroxetine hydrochloride, citalopram hydrobromide, sertraline hydrochloride, fluoxetine hydrochloride, fluvoxamine maleate, venlafaxine hydrochloride, phenelzine sulfate salt, entacapone, GBR 12935 dihydrochloride, nisoxetine hydrochloride, hydrocortisone, and decynium-22 were purchased from Sigma-Aldrich (St. Louis, USA). Pierce™ BCA Protein Assay Kit was purchased from Thermo Fisher Scientific (Waltham, United States). All other chemicals were of analytical grade.

### Primary trophoblast cell isolation, purity evaluation, and culture

Human term placentas were collected from uncomplicated pregnancies after elective caesarian section (gestation week 39–40) at the University Hospital in Hradec Kralove, Czech Republic. All donating women signed a written informed consent form. Experiments were performed following the Declaration of Helsinki and with approval of the University Hospital Research Ethics Committee (201006 S15P). Clinical characteristics of pregnancies involved in the study are listed in Supplementary Table S1.

PHT cells were isolated from the term placentas as previously described [31]. Briefly, the tissue was subjected to enzymatic digestion three times with 0.25% trypsin (Gibco; Thermo Fisher Scientific, USA) and 300 IU/ml deoxyribonuclease I (Sigma Aldrich, USA) at 37 °C for 30 min. CTBs were isolated using Percoll (Sigma Aldrich, USA) density gradient separation. Cell purity was evaluated by 1-h labeling with specific cell marker antibodies at room temperature and subsequent flow cytometry analysis, as previously described [31]. The following antibodies (supplied by Novus Biologicals, USA) were used: anti-cytokeratin 7 (AF 488®), anti-vimentin (AF 647®), anti-von Willebrand Factor (AF 647®). At least 10,000 cells were scanned by a SA3800 Spectral Analyzer (Sony Biotechnology, USA) and acquired data were analyzed using the FCS Express package from De Novo Software. Results of cell purity analyses are shown in Supplementary Table S2.

The isolated cells were cultured in high glucose Dulbecco's Modified Eagle Medium (DMEM) supplemented with GlutaMAX™ (Gibco; Thermo Fisher Scientific, USA), and enriched with 10% FBS and 1% Penicillin—10,000 U/Streptomycin—10 mg/ml (Sigma Aldrich, USA). The cells were incubated at 37 °C, with 5% CO_2_ + 95% air for 12 h (PHT-CTB) or 72 h (PHT-STB, with daily change of medium).

### BeWo cell culture

Human choriocarcinoma-derived BeWo cells obtained from the European Cell Culture Collection (Salisbury, United Kingdom) were cultured in Ham F-12 medium enriched with 10% fetal bovine serum (FBS) and grown at 37 °C, with 5% CO_2_ + 95% air and no antibiotics. To assess effects of cell differentiation, cells were seeded in media containing either 0.1% DMSO (control; BeWo-CTB) or 20 µM forskolin (in 0.1% DMSO; BeWo-STB) for 48 h with daily change of the medium.

### Cell differentiation determination by measuring hCG secretion

To check the success of PHT and BeWo cells’ spontaneous and forskolin-induced differentiation to STB, respectively, samples of culture medium were collected during their cultivation in both CTB and STB stages. The concentration of human Chorionic Gonadotropin (hCG), a marker of villous trophoblast differentiation [32], was determined using the Human hCG (intact) using enzyme-linked immunosorbent assay (ELISA) Kit (Sigma Aldrich, USA), according to the manufacturer’s instructions.

### RNA isolation and protein extraction

RNA was isolated from cells using TRI reagent (Molecular Research Centre, USA), according to the manufacturer’s instructions. RNA purity and concentration were evaluated by measuring absorbance with a NanoDrop™ spectrophotometer (Thermo Fisher Scientific, USA). For protein extraction, cells were lysed in cell lysis buffer [20 mM Tris, 150 mM NaCl, 12.8 mM EDTA, 1 mM EGTA, 4.2 mM Na-pyrophosphate, 1 mM Na_3_VO_4_, 17 µM Triton, supplemented with Protease Inhibitor Cocktail (P8340; Sigma Aldrich, USA); pH adjusted to 6.8]. Protein concentrations of samples were measured using a Pierce™ BCA Protein Assay Kit (Thermo Fisher Scientific, USA).

### Reverse transcription and gene expression analysis

Reverse transcription was performed using an iScript Advanced cDNA Synthesis kit and a T100™ Thermal Cycler (both from Bio-Rad, USA), according to the manufacturer’s instructions. qPCR reaction was performed on the QuantStudio™ 6 Flex Real-Time PCR System (Applied Biosystems; Thermo Fisher Scientific, USA). 12.5 ng portions of obtained cDNA were amplified in 384-well plates (5 µl/reaction) using TaqMan™ predesigned assays and Universal Master Mix II without UNG (all supplied by Thermo Fisher Scientific, USA) as previously described [31]. Expression levels of the following genes were estimated: *NET/SLC6A2* (Hs00426573_m1), *DAT/SLC6A3* (Hs00997374_m1), *SERT/SLC6A4* (Hs00984349_m1), *OCT3/SLC22A3* (Hs01009571_m1), and *hCG/CGB3* (Hs00361224_gH). For this, transcripts of the genes were amplified in triplicate, using thermal programs recommended by the manufacturer, and quantified by the ΔΔCt method with *YWHAZ* (Hs01122445_g1) and *B2M* (Hs00187842_m1) as reference genes, as previously described [33].

### Western blotting analysis of protein expression

Portions of cell lysates (with 40 μg total protein) were mixed with loading buffer containing a reducing agent [34]. Proteins in them were denatured by incubation at 96 °C for 5 min, then separated by SDS-PAGE on 12.5% polyacrylamide gel at 130 V and transferred to polyvinylidene fluoride (PVDF) membrane (SERVA, DE) using a Trans-Blot® Turbo™ Transfer System (Bio-Rad, USA). The membrane was blocked by incubation with 5% BSA for 1 h at room temperature then washed with TBS-T buffer. The membrane was then incubated overnight at 4 °C with primary rabbit antibodies raised against NET/SLC6A2 (1:500, Cat. No. Ab41559; Abcam, UK), DAT/SLC6A3 (1:1,000, Cat. No. D6944; Sigma Aldrich, USA), SERT/SLC6A4 (1:500, Cat. No. SAB4200039; Sigma-Aldrich, USA) and OCT3/SLC22A3 (1:10,000, Cat. No. Ab124826; Abcam, UK). The membranes were subsequently incubated with Swine anti-rabbit Immunoglobulins/HRP (1:10,000, Cat. No. P0217; Dako, DK) for 1 h at room temperature. Protein bands were labelled using Amersham ECL Prime Western Blotting Detection Reagent (Cytiva, UK), then visualized and quantified densitometrically by a ChemiDoc^TM^ MP Imaging system (Bio-Rad, USA). Ponceau S (Sigma Aldrich, USA) staining was used to check that protein loadings of the samples were roughly equal. Representative original, full-length blots can be found in Supplementary figure S1.

### Monoamine uptake by placental cells

In vitro uptake studies were performed with a 96-well plate setup. 1 × 10^5^ of PHT cells were seeded in Nunc™ Delta-treated surface plates (Nunc; Thermo Fisher Scientific, USA) and 0.2 × 10^5^ of BeWo cells were seeded in TPP culture plates (Techno Plastic Products AG, CH). Uptake of the focal monoamines was then initiated by incubation with 1 µCi/ml ^3^H-serotonin, ^3^H-norepinephrine, and ^3^H-dopamine in Opti-MEM™ (Gibco; Thermo Fisher Scientific, USA) in the presence of 1 mM ascorbic acid (to prevent monoamine oxidation), 100 µM phenelzine (MAO-A inhibitor), and 0.5 µM entacapone (COMT inhibitor; used in norepinephrine and dopamine, but not serotonin, uptake assays). At specific time points (1, 5, 15, 30, and 60 min), uptake was halted by two steps of cell washing with ice-cold Dulbecco′s Phosphate Buffered Saline (Sigma Aldrich, USA) at 4˚C. In uptake inhibition assays, cells were initially incubated for 10 min in Opti-MEM™ containing either 0.1% DMSO (control) or the inhibitors/drugs of interest (in 0.1% DMSO). The following specific inhibitors were used: 100 µM paroxetine, 20 nM nisoxetine, 50 nM GBR 12935, 100 µM decynium-22, and 100 µM cortisol. On the other hand, antidepressant drugs were used at physiologically achievable plasma concentrations (0.3 to 3 µM). Subsequently, the cells were then incubated in the presence of ^3^H-serotonin, ^3^H-norepinephrine, or ^3^H-dopamine, with or without the selected inhibitors/antidepressants, and intracellular levels of the labeled monoamines were measured 15 min later. In Na^+^-dependency assays, Opti-MEM™ was replaced by Na^+^ buffer (140 mM NaCl, 5.4 mM KCl, 1.8 mM CaCl_2_ 2H_2_O, 0.8 mM MgSO_4_ 7H_2_O, 5 mM glucose, 25 mM Tris; pH 7.4) and a corresponding Na^+^-free buffer (containing 140 mM N-methyl-D-glucamine instead of NaCl). PHT and BeWo cells were lysed for radioactivity measurement in 0.5 M KOH and 0.02% SDS, respectively. Their intracellular monoamine concentrations were determined by liquid scintillation counting with a Tri-Carb 2910 TR instrument (Perkin Elmer, USA) and normalized against the protein content (determined using the Pierce™ BCA Protein Assay Kit). Uptake is reported in pmol/mg protein or as percentages of the uptake by controls (at room temperature, in the presence of Na^+^).

### Statistical analysis

Differences between samples in transcript or protein expression levels were evaluated using paired t-tests. Differences in levels of monoamines were analyzed using mixed-effects analysis or One-way ANOVA, followed by Dunnett’s multiple comparisons test. * *p* ≤ 0.05, ** *p* ≤ 0.01, *** *p* ≤ 0.001. GraphPad Prism version 9.2.0 was used for all data analysis and graphical presentation.

## Results

### Expression and secretion of syncytial marker hCG

**Fig. 1:**
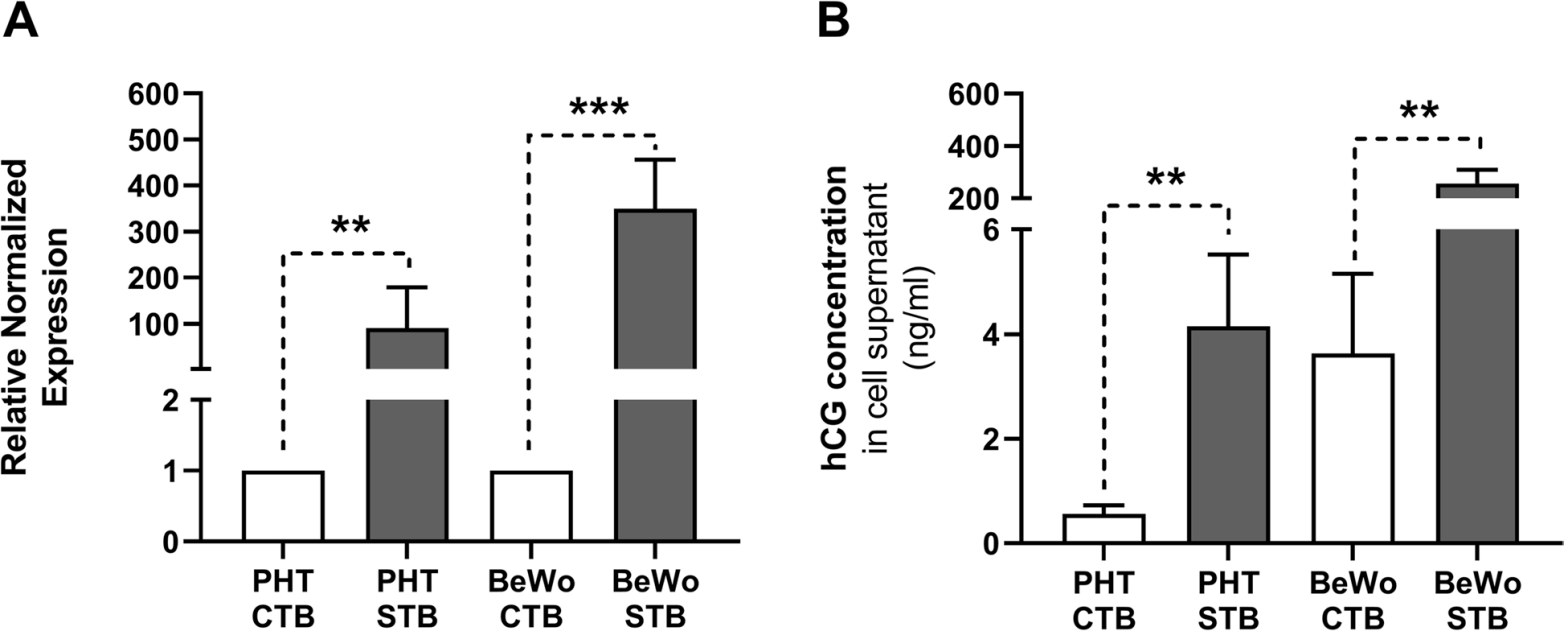
Cellular expression and secretion of hCG as a marker of trophoblast differentiation. Differences in hCG expression and secretion, respectively measured by quantitative PCR (**A**) and ELISA (**B**), between undifferentiated (CTB) and differentiated (STB) cells. Presented data are means ± SD (*n* ≥ 4), with indications of statistical significance obtained from paired t-tests: **p ≤ 0.01, ***p ≤ 0.001

hCG gene expression and protein secretion were used to evaluate the syncytialization of PHT cells (spontaneously after 72 h) and BeWo cells (following 48-h treatment with 20 µM forskolin). Transcript levels of *hCG* rose 91- and 350-fold in the PHT and BeWo cells, respectively (Fig. 1A). Corresponding increases in protein-level concentrations of the soluble hormone in culture medium were also recorded in the PHT-STB and BeWo-STB cells (Fig. 1B), confirming that successful syncytialization occurred in both cellular models.

### Changes in monoamine transporter expression associated with trophoblast differentiation 

**Fig. 2:**
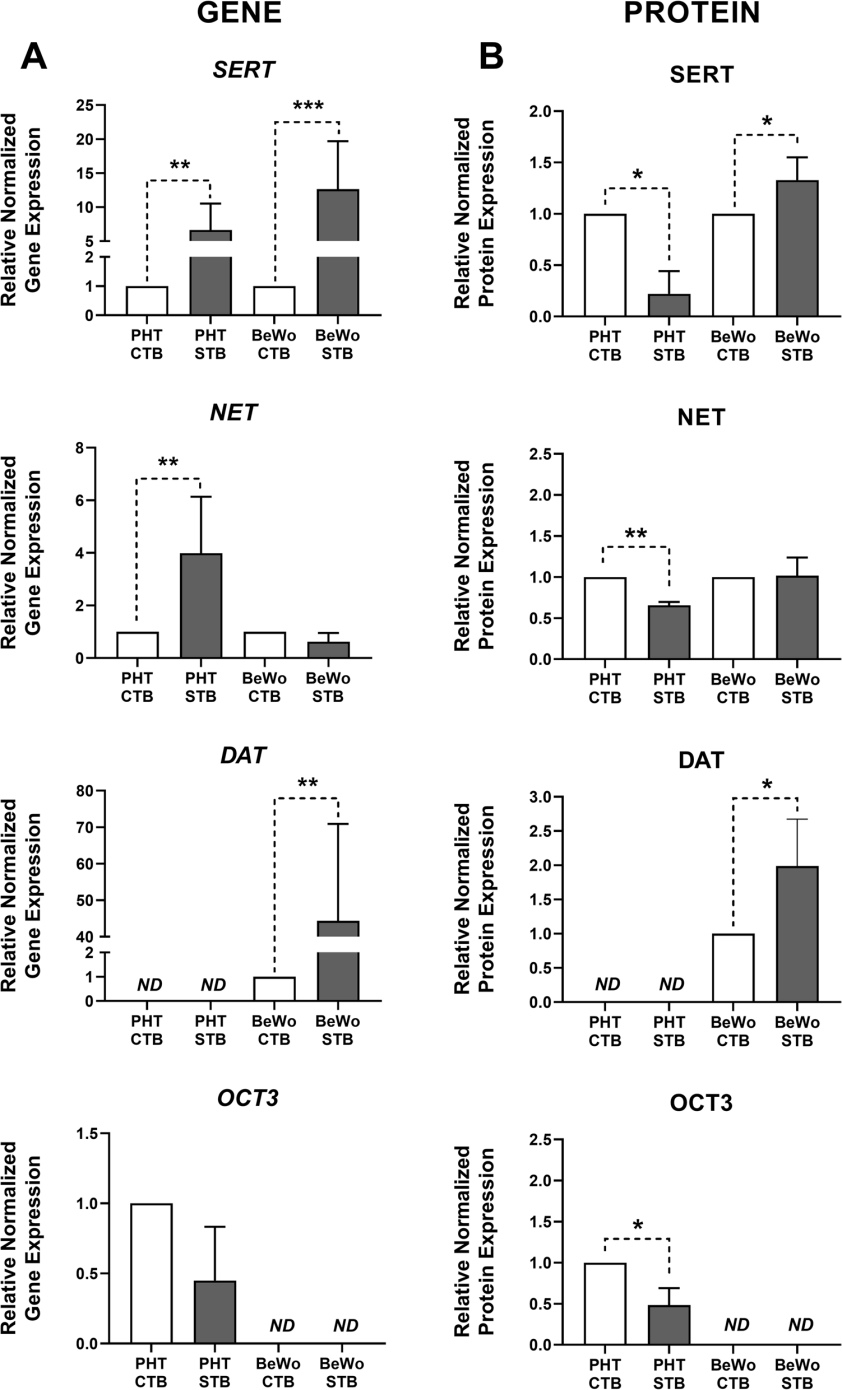
Gene and protein expression of the monoamine transporters in placental cells. Results of quantitative PCR (**A**) and Western blot (**B**) analysis of SERT, NET, DAT, and OCT3 showing fold-differences in expression levels of the genes between undifferentiated (CTB) and differentiated (STB) PHT and BeWo cells. Gene expression results were normalized to the geometric mean of *YWHAZ* and *B2M* expression, whereas protein expression was normalized to the total protein, visualized by Ponceau S staining. Presented data are means ± SD (*n* = 4), with indications of statistical significance obtained from paired t-tests: *p ≤ 0.05, **p ≤ 0.01, ***p ≤ 0.001, *ND* = not detected

Analysis of transcript- and protein-level expression of key monoamine transporters (SERT, NET, DAT, and OCT3) revealed significant differences in their expression profiles between PHT and BeWo cells, at both basal level and following trophoblast differentiation in vitro (Fig. 2). Both cellular models expressed SERT and NET, but no expression of DAT was detected in PHT cells and no expression of OCT3 in BeWo cells (Fig. 2A and B). For original uncropped western blot images see Figure S1 in Supplementary file.

Importantly, between-model alterations in monoamine transporter expression were also noted following trophoblast differentiation. Forskolin-induced trophoblast differentiation in BeWo cells was associated with a pronounced upregulation of SERT gene and protein expression, and no change in NET (Fig. 2A and B). On the other hand, while *SERT* and *NET* genes were upregulated during the spontaneous syncytialization process in PHT cells (Fig. 2A), SERT and NET protein expression showed significant downregulation (Fig. 2B). Similarly, DAT expression was significantly upregulated in BeWo-STB both at gene and protein level (Fig. 2A and B). Lastly, expression of OCT3 protein decreased significantly during differentiation in PHT cells (Fig. 2B).

### Effect of trophoblast differentiation on in vitro monoamine uptake

**Fig. 3:**
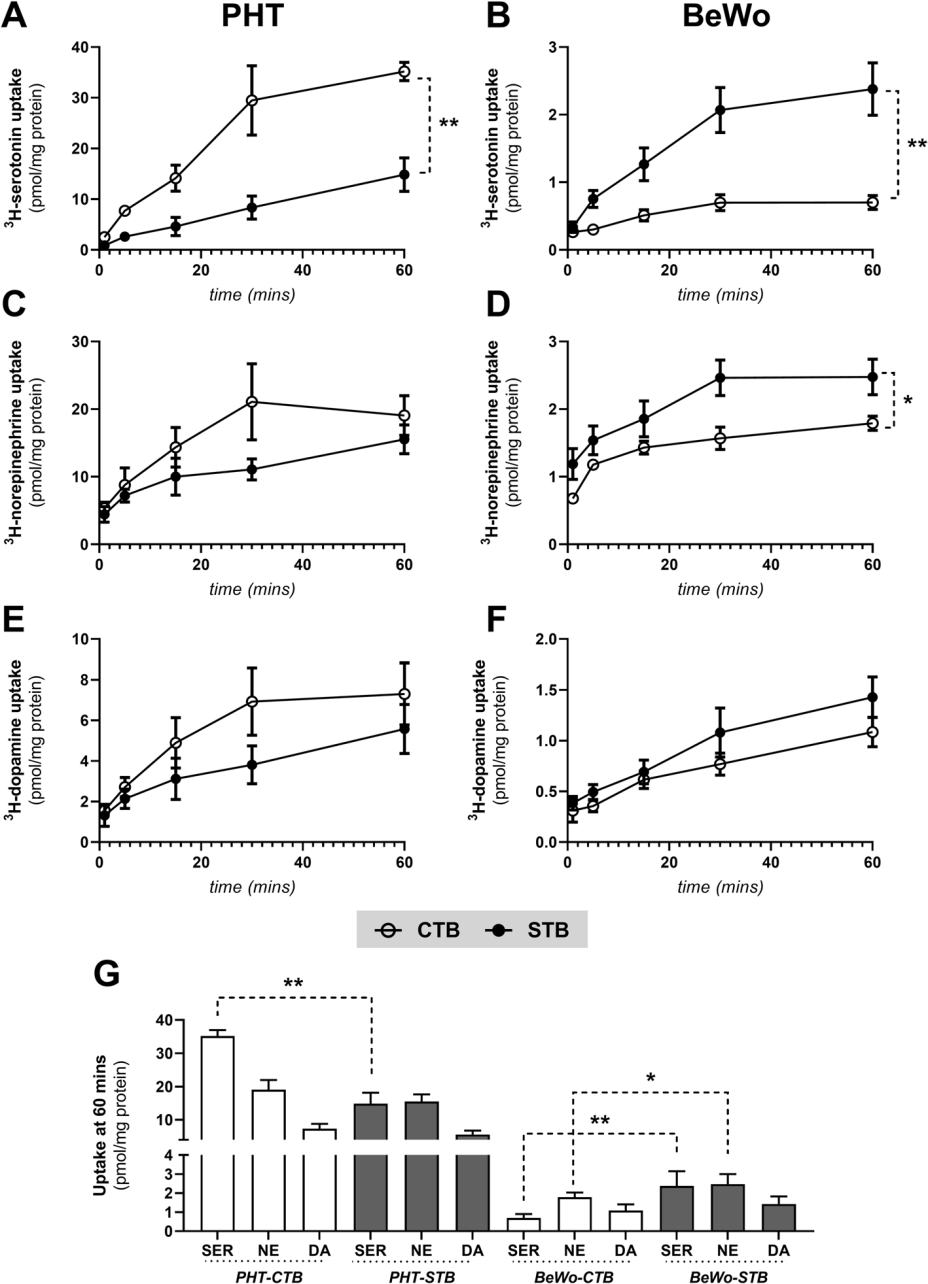
Time courses of monoamine uptake in PHT and BeWo cells. Monitoring of serotonin (**A**, **B**), norepinephrine (**C**, **D**), and dopamine (**E**, **F**) uptake over time showed that they were roughly linearly transported for up to 30 min, equilibrium was reached within 60 min, and uptake rates differed between both the two cellular models and differentiation states (**G**). Presented data are means ± SD (*n* ≥ 4), with indications of statistical significance obtained from mixed-effects analysis: *p ≤ 0.05, **p ≤ 0.01

Analyses of time courses of serotonin, norepinephrine, and dopamine uptake by PHT and BeWo cells showed that uptake of all three monoamines increased for 30 min then reached equilibrium values within a further 30 min (Fig. 3). However, there was clear evidence of regulatory differences during their trophoblast differentiation. The transporters’ activity was apparently down-regulated during spontaneous cell differentiation in PHT cells (Fig. 3A, C and E), while (in stark contrast) forskolin-stimulation of BeWo cells resulted in significant increases in their serotonin levels (Fig. 3B) and, to a lesser extent, norepinephrine levels (Fig. 3D). In addition, total monoamine uptake capacity at equilibrium of PHT cells was several times higher than that of BeWo cells (Fig. 3G).

### Characterization of mechanisms mediating monoamine transport in PHT and BeWo cells

To identify the transport systems responsible for uptake of each of the three monoamines in placental cells we investigated effects of temperature, NaCl gradient, and classical monoamine transporter inhibitors on their uptake rates. The inhibitors were: paroxetine (SERT and OCT3 inhibitor), nisoxetine (NET inhibitor), GBR 12935 (DAT inhibitor), decynium-22 and cortisol (OCT3 inhibitors). The concentrations chosen were based on previously published literature reporting the inhibitors’ degrees of selectivity for the transporters [16, 35, 36].

Observed rates of temperature- and Na^+^-dependent monoamine uptake are shown in Table 1. Temperature-dependent uptake accounted for > 80% of the total uptake of serotonin by PHT cells, indicating the presence of a specific transport mechanism. In contrast, uptake of norepinephrine in PHT cells was significantly less affected by incubation at 4 °C (relative to the rate at room temperature), with the transporter-mediated mechanism accounting for 47.6% and 40.2% of total uptake in the CTB and STB stages, respectively. Similarly, temperature-independent dopamine uptake dominated in PHT cells, accounting for more than 80% of the total. In BeWo cells, the apparent contribution of carrier-mediated transport was generally lower for all monoamines, and only significant in the STB stage.

**Table 1:** Effects of temperature and Na^+^ gradient on the uptake of monoamines by placental cells

		**Uptake at 4 °C**			**Uptake in absence of Na**^**+**^		
**Monoamine**	**Cell model**	**mean (%)**	**SD**	***p* value**	**mean (%)**	**SD**	***p* value**
Serotonin	PHT-CTB	15.56	11.50	0.0014 **	19.41	10.19	0.0024 **
	PHT-STB	12.96	6.87	0.0001 ***	23.36	6.94	0.0023 **
	BeWo-CTB	75.56	13.70	0.0201 *	48.09	15.72	0.0022 **
	BeWo-STB	32.46	5.15	0.0078 **	35.36	10.97	0.0049 **
Norepinephrine	PHT-CTB	52.40	14.29	0.1336	65.63	22.82	0.0782
	PHT-STB	59.76	6.61	0.1306	82.23	18.19	0.1960
	BeWo-CTB	106.60	14.58	0.6296	-	-	-
	BeWo-STB	85.95	20.15	0.287	95.37	29.54	0.4657
Dopamine	PHT-CTB	80.70	12.54	0.0925	71.19	31.19	0.4075
	PHT-STB	91.07	28.80	0.8170	72.44	39.95	0.3676
	BeWo-CTB	100.10	9.04	0.9585	-	-	-
	BeWo-STB	70.62	21.04	0.0663	75.48	18.97	0.960

Values shown are percentages of monoamine uptake at 4 °C/in the absence of Na^+^ relative to uptake at room temperature and in the presence of Na^+^ (and *p* values of the differences). Presented data are means ± SD (*n* ≥ 4). The statistical significance of differences was evaluated using paired t-tests: *p ≤ 0.05, **p ≤ 0.01, ***p ≤ 0.001

Subsequently, to identify the carrier-mediated transport’s dependence on a Na^+^ gradient and sensitivity to inhibitors, the specific uptake for each monoamine was estimated by calculating the difference between its transport at room temperature and 4 °C (except for norepinephrine and dopamine in BeWo-CTB cells, for which all recorded uptake was transporter-independent). We found that serotonin was the only monoamine whose uptake was significantly lower in the absence of a Na^+^ gradient, in both cellular models (Table 1). In addition, uptake of norepinephrine showed some Na^+^-dependence in PHT cells, but not BeWo cells. Likewise, dopamine cellular transport was predominantly via a Na^+^-independent mechanism.

**Fig. 4:**
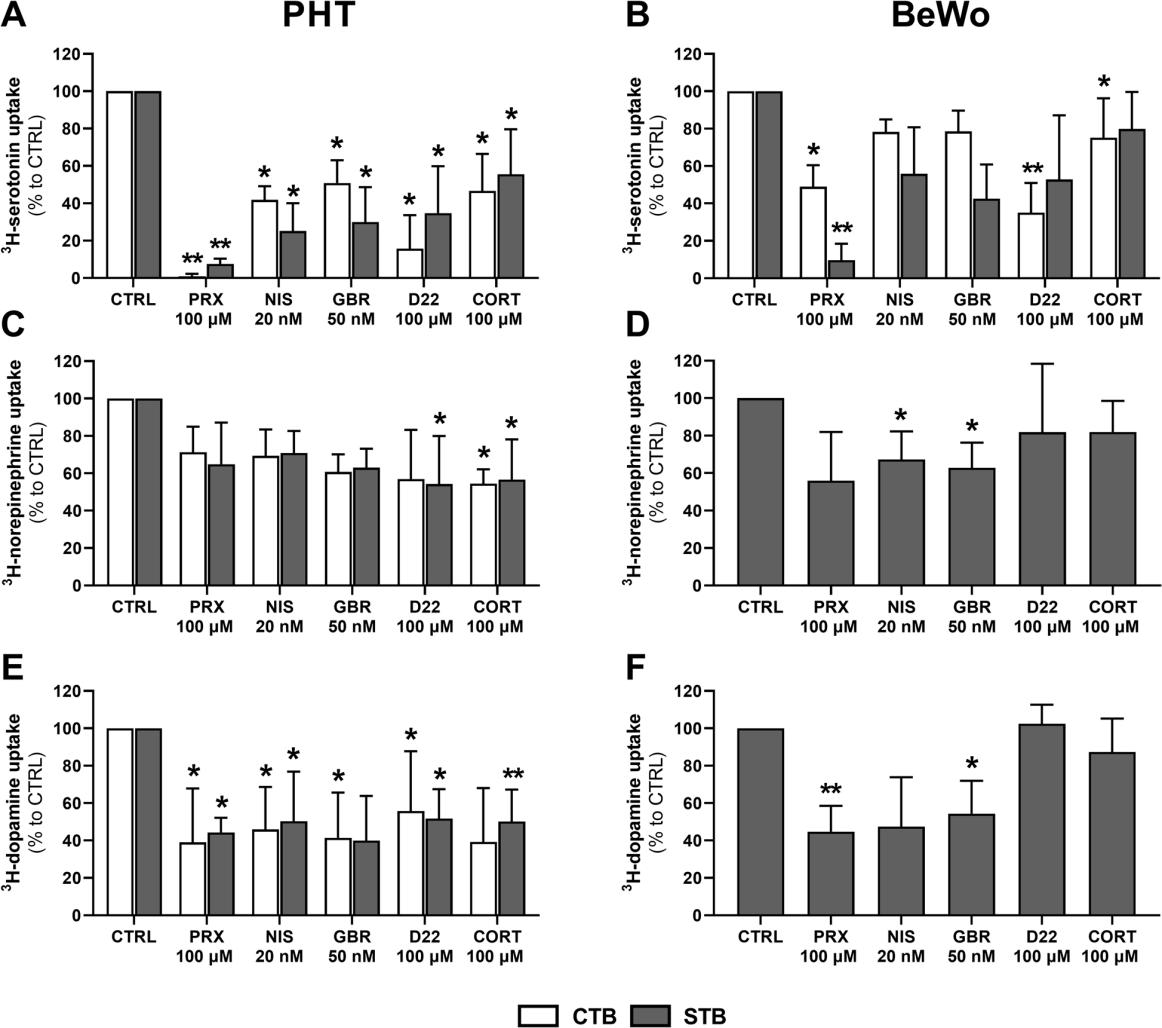
Differential sensitivity of monoamine transport systems to inhibitors in PHT and BeWo cells. Uptake of serotonin (**A**, **B**), norepinephrine (**C**, **D**), and dopamine (**E**, **F**) recorded in the presence of 100 µM paroxetine (PRX), 20 nM nisoxetine (NIS), 50 nM GBR 12935, 100 µM decynium-22 (D22), and 100 µM cortisol (CORT). Values are percentages of the recorded uptake by inhibitor-free controls (CTRL). Presented data are means ± SD (*n* ≥ 4), with indications of statistical significance obtained from paired t-tests compared to CTRL: *p ≤ 0.05, **p ≤ 0.01

After deducting the temperature-independent transport (at 4 °C), we next compared effects of various inhibitors on the monoamines’ transporter-mediated uptake by placental cells (Fig. 4). The results show that paroxetine, nisoxetine, GBR 12935, decynium-22, and cortisol are significant inhibitors of serotonin (Fig. 4A) and dopamine (Fig. 4E) uptake by PHT cells, suggesting that these monoamines share common transport systems. In contrast, norepinephrine accumulation in these cells was only inhibited, weakly, by decynium-22 and cortisol (Fig. 4C). In contrast, paroxetine significantly affected serotonin (Fig. 4B) and dopamine (Fig. 4F) uptake by BeWo cells, while GBR 12935 inhibited norepinephrine (Fig. 4D) and dopamine (Fig. 4F) uptake. In the BeWo-CTB stage, we also observed an effect of decynium-22 on serotonin uptake (Fig. 4B). Collectively, these results indicate that PHT and BeWo cells have transport mechanisms with distinct features for all three monoamines.

### Effects of antidepressant drugs on serotonin uptake by placental cells

**Fig. 5:**
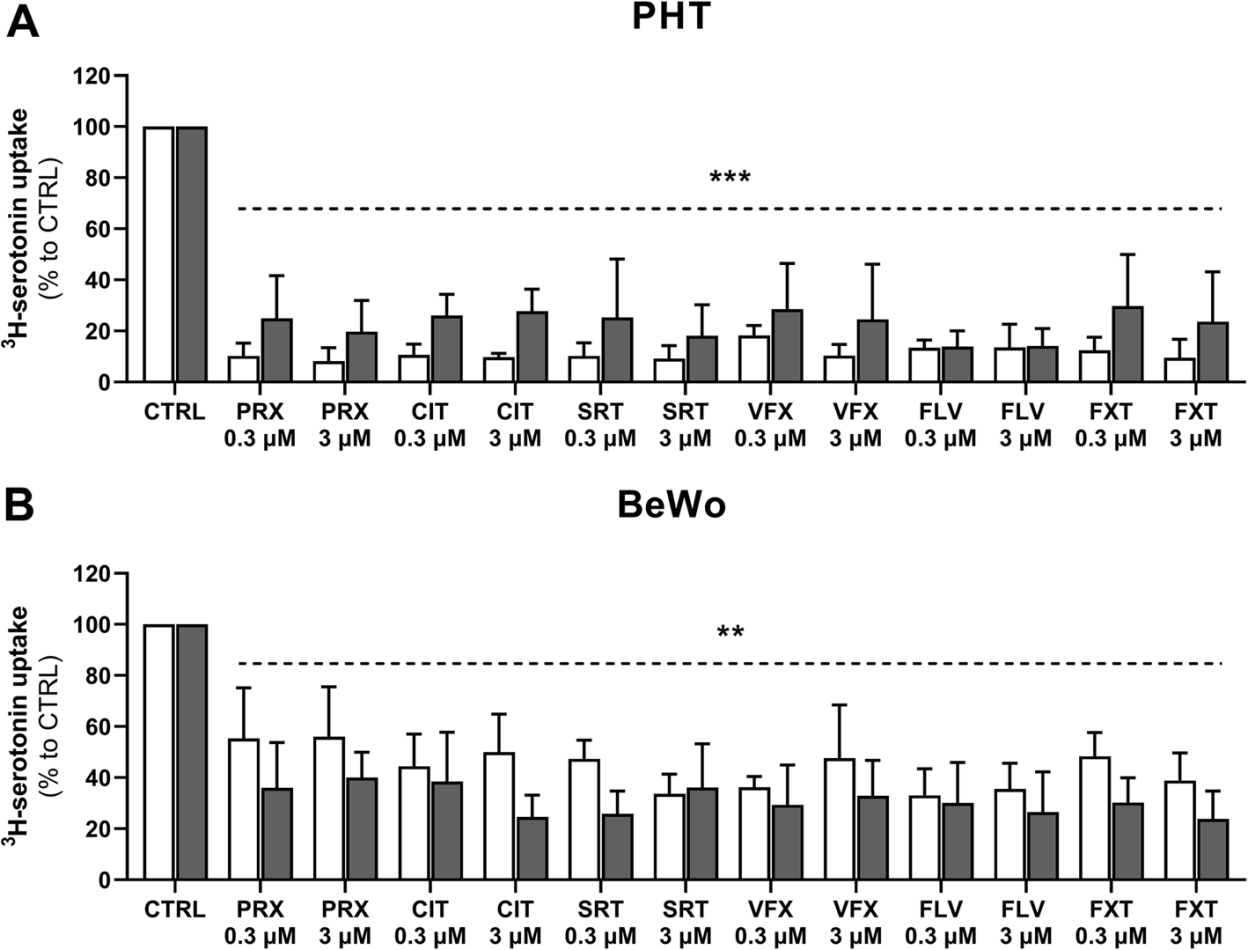
Inhibition of human placental cells’ serotonin uptake by antidepressant drugs. Uptake of serotonin by PHT (**A**) and BeWo (**B**) cells in the presence of selected antidepressant drugs: paroxetine (PRX), citalopram (CIT), sertraline (SRT), venlafaxine (VFX), fluvoxamine (FLV), and fluoxetine (FXT) at physiologically achievable plasma concentrations (0.3 to 3 µM). Values shown are percentages of uptake by inhibitor-free controls (CTRL). Presented data are means ± SD (*n* ≥ 4), with indications of statistical significance shown as a dotted line and obtained from one-way ANOVA summary compared to CTRL: **p ≤ 0.01, ***p ≤ 0.001

Next, we investigated effects of selective serotonin reuptake inhibitors (paroxetine, citalopram, sertraline, fluvoxamine, and fluoxetine) and a selective serotonin/norepinephrine reuptake inhibitor (venlafaxine) on cellular serotonin accumulation. At physiologically achievable plasma concentrations (0.3 to 3 µM) all tested antidepressants affected serotonin uptake by PHT and BeWo cells, with variations in potency between both the cell models and cell stages (Fig. 5). In CTB and STB stages of PHT cells, initial uptake rates of serotonin were inhibited by about 13 and 21%, respectively (Fig. 5A). However, serotonin accumulation was less strongly affected in BeWo cells (by about 44 and 32% in the CTB and STB stages, respectively; Fig. 5B). Thus, serotonin uptake was more susceptible to inhibition by the selected antidepressants in the CTB stage of PHT cells and the STB stage of BeWo cells. No significant differences in any of the inhibitors’ potency at the two concentrations used were observed.

## Discussion

**Fig. 6:**
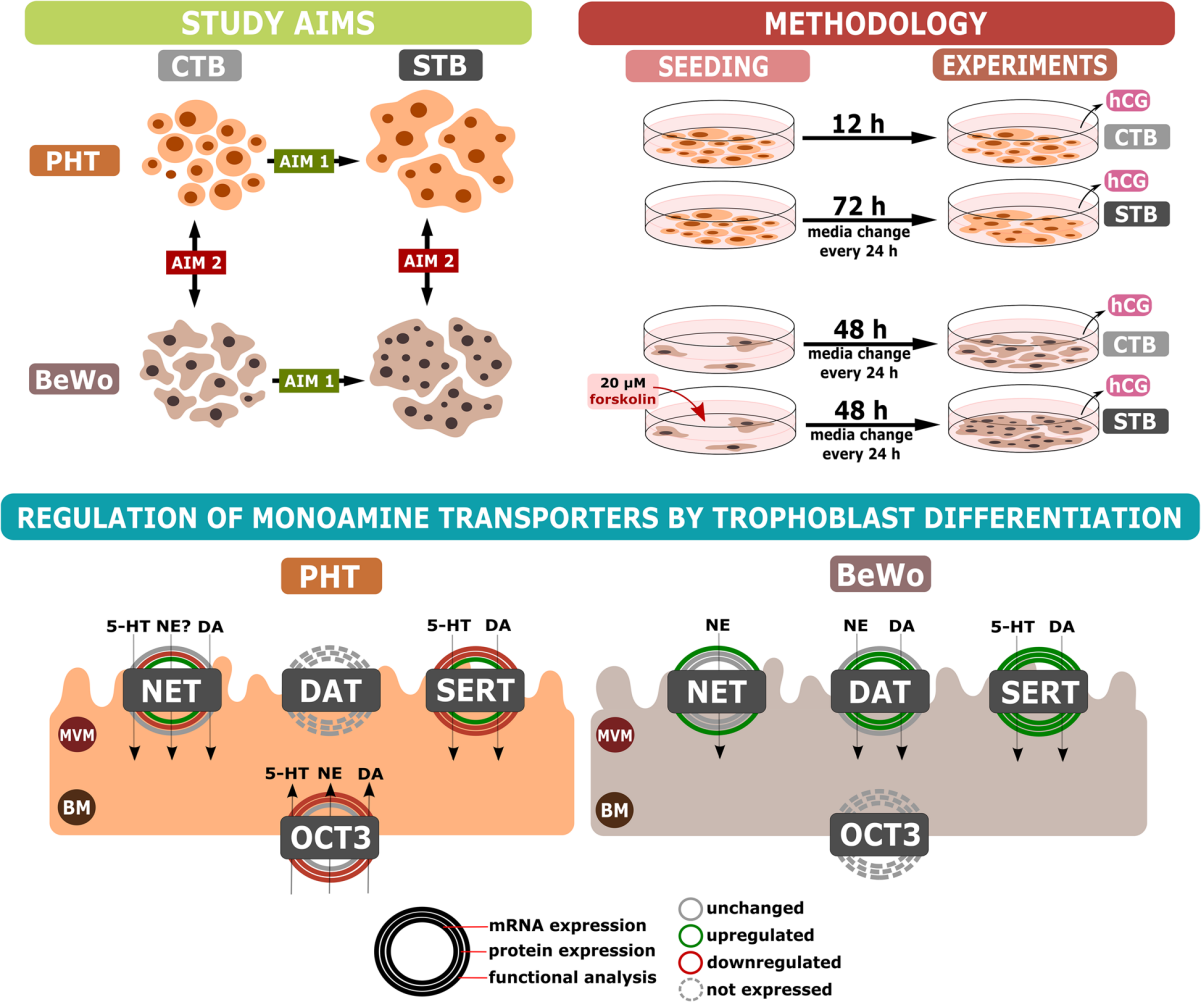
Summary of changes in placental monoamine uptake associated with trophoblast differentiation. Aims of the study were to characterize changes in monoamine transporter expression and function associated with trophoblast differentiation in vitro, and identify the optimal placental cell model for monoamine regulation studies. Isolated primary trophoblast cells from human term placenta (PHT) and the BeWo human choriocarcinoma cell line were used in the study. PHT cells differentiated spontaneously during a 72-h culture period, and BeWo cells’ differentiation was induced by incubation with 20 µM forskolin for 48 h. *hCG* transcripts in cells and hCG protein levels in their media were measured as molecular markers of trophoblast differentiation, and changes in both expression and function of monoamine transporters associated with the process were characterized. The results show that the process is accompanied by significant regulatory control of the transporters’ functionality and that cells derived from choriocarcinoma-derived lines, such as BeWo, may not be suitable models for studying placental monoamine transport due to both transcriptional and functional differences. Abbreviations: BM – basal membrane; CTB – cytotrophoblast; DA – dopamine; DAT – dopamine transporter; hCG – human chorionic gonadotropin; MVM – microvillous membrane; NE – norepinephrine; NET – norepinephrine transporter; OCT3 – organic cation transporter 3; PHT – primary trophoblast cells; SER – serotonin; SERT – serotonin transporter; STB – syncytiotrophoblast

In this study we detected changes in the transcriptional and functional regulation of monoamine transporters associated with phenotypic transitioning from CTBs to a STB layer using multiple techniques and two complementary placental in vitro models (Fig. 6). The findings extend knowledge of monoamine regulation, which has increasingly recognized importance in prenatal development and fetal programming [5, 37]. They also have important implications for the validity of models used to address the processes involved.

Previous studies have shown that the trophoblast differentiation process is associated with simultaneous induction and repression of numerous genes that encode functionally related proteins [38, 39]. We have also recently found that several placental membrane proteins responsible for the transport of nutrients and xenobiotics are differentially expressed in the CTB and STB stages [33]. Here, we show that the expression of key monoamine transporters (SERT, NET, and DAT) is significantly altered during, and after, placental cell differentiation. In highly purified PHT cells this process occurred spontaneously via the cAMP signaling pathway, which further activates cell fusion effectors [19]. In contrast, syncytium formation in BeWo cells is induced by external stimulation through the action of cAMP inducers such as forskolin [30]. However, while some aspects of the syncytialization process are putatively similar in these two models, there are distinct reported differences between them in formation of the syncytium, its epigenetic status, and regulatory elements [21, 40]. Accordingly, in this study we found that while *SERT* and *NET* mRNA expression was upregulated in PHT cells upon cell differentiation, the protein expression was downregulated. In contrast, SERT mRNA and protein expression in BeWo cells significantly increased with syncytialization, consistent with previous reports in the JAr choriocarcinoma cell line, in which the cited authors postulate that cAMP induction stimulates de novo synthesis of SERT protein [41]. A recent study highlights SERT and DAT as targets of transcriptional regulators of trophoblast identity [42]; thus, we speculate that in PHT cells these two genes exhibit post-transcriptional regulation during the differentiation process.

Apart from the transcriptional profile, our findings further highlight the association of trophoblast differentiation with differences in the cells’ accumulation of monoamines. Uptake of serotonin in PHT cells was higher in the undifferentiated CTB stage, whereas uptake of serotonin and norepinephrine in BeWo cells was upregulated in the differentiated STB cells. Moreover, comparison of uptake kinetics over 60 min showed that total monoamine uptake was several-fold higher in PHT cells than in BeWo cells. These differences can be explained by differences in monoamine transport profiles between the two cell models. Most prominently, unlike PHT cells, the BeWo cell line does not express OCT3, a key transporter responsible for uptake of serotonin from the fetal circulation [16]. This deficiency was also previously reported in JEG-3, another choriocarcinoma-derived cell line [43]. We also found that SERT, NET, and OCT3 expression in PHT cells was higher in the CTB stage. OCT3 expression results are in accordance with previous detection of prominent OCT3 signals in the cytoplasm of cytotrophoblasts in all three trimesters [44]. As OCT3 is a high-capacity transporter for serotonin [16], we suggest that it is the main transporter accounting for PHT cells’ higher accumulation capacity than BeWo cells. Moreover, the lower serotonin uptake in the STB stage may reflect the lower SERT, NET, and OCT3 protein expression observed in this cell stage.

Remarkably, we found that BeWo cells express DAT, which we did not detect in PHT cells, and according to current literature is not expressed in human placental tissue [15]. To the best of our knowledge, this is the first report of DAT expression in any choriocarcinoma-derived cell line. As BeWo cells were established from metastatic choriocarcinoma isolated from the brain, it is intriguing to speculate that this feature is the outcome of genetic drift resulting in a more favorable phenotype for cell proliferation and growth. Accordingly, dopamine is reportedly a proliferation and differentiation inducer of neural cells [45], and contributor to the growth of certain brain tumors [46]. However, the relevance of DAT expression in these cells requires further investigation.

Next, we aimed to characterize the transport systems responsible for the monoamine uptake by placental cells. In contrast to membrane preparations isolated from the human placenta, which have a predominantly active uptake mechanism for monoamines [15], we detected substantial accumulation of norepinephrine and dopamine by placental cells incubated at 4 °C. As membrane transport proteins are inhibited in these conditions, this suggests either substrate binding to the cellular membrane or a contribution of passive diffusion. Accordingly, passive accumulation of these monoamines has previously been reported in other nonneuronal cells, such as astrocytes, pheochromocytoma, and renal epithelial cells [47–49]. Moreover, after subtracting the nonspecific uptake (at 4 °C), we only detected Na^+^-dependent transport of serotonin. These patterns are similar to those previously reported for JAr cells, in which no Na^+^-dependent norepinephrine and dopamine uptake has been observed [50]. As the function of NET, and DAT, depends on a Na^+^ gradient, the cited authors postulated that this indicates a possible loss of transporter function in JAr cells [15]. However, in PHT cells, while the uptake was independent of Na^+^, dopamine uptake sensitivity to the selective inhibitors was similar to that of serotonin, indicating that dopamine and serotonin may have certain common transport mechanisms in these cells. This phenomenon has also been observed in other nonneuronal cells such as platelets [51]. In addition, classic OCT3 inhibitors (decynium-22 and cortisol) significantly affected uptake of dopamine and norepinephrine in PHT cells. Collectively, these results suggest that norepinephrine and dopamine uptake in PHT cells is mediated by a combination of active transport (mainly via OCT3 as a Na^+^-independent transporter) and passive diffusion.

With depression rates on the rise, the latest data from six European countries suggest that more than 10% of pregnant women are exposed to antidepressant drugs during the course of their pregnancy [52]. However, these drugs can cross the placental barrier and several epidemiological studies have detected a link between antidepressant use during pregnancy and impairment of fetal brain development and programming [53, 54]. We have recently reported that serotonin reuptake inhibitors can affect placental serotonin homeostasis by inhibiting both SERT and OCT3, thus resulting in suboptimal serotonin concentrations in the fetoplacental unit [55]. In this study we further found that, at pharmacologically relevant drug concentrations, a wide range of selective serotonin reuptake inhibitors impair serotonin cellular accumulation in both PHT and BeWo cells. Similar observations in PHT cells have also been recently reported, with half-maximal inhibitory concentrations in the 2–11 nM range [56], highlighting these drugs’ potentially negative effects on cellular serotonin processing. However, the extent of serotonin uptake inhibition by these drugs differs between PHT and BeWo cells, probably due to the lack of OCT3 (which is also sensitive to selective serotonin uptake inhibitors [57]). Overall, these findings suggest that PHT cells (but not BeWo cells) provide an appropriate model to investigate cellular effects of psychotropic drugs on the placental serotonin system.

The downregulated monoamine uptake with trophoblast differentiation was surprising since the STB has traditionally been associated with the placental transport function. One important aspect to consider is the potential active participation of monoamine transporters in trophoblast differentiation. Regulation of intracellular levels of monoamines may contribute to the physiological modulation of cellular functions such as division and differentiation, as described in other systems [27–29]. Moreover, contrary to previous beliefs, the CTB stage has recently been recognized as the site with the highest metabolic activity in the placenta [58]. In addition, it reportedly exclusively regulates placental fatty acid uptake and metabolism, refuting the concept that nutrient processing is restricted to the STB layer [24]. Thus, the undifferentiated CTBs may also participate in nutrient transport, and the role of the polyspecific OCT3 in these cells warrants further attention.

A major strength of this study is the use of PHT cells isolated from the human term placenta. Various other models may be suitable for studying some developmental processes of trophoblasts, but these are the only effective models for studying STB formation [19]. Importantly, while BeWo cells have been shown to share some characteristics of trophoblasts [30], we have shown that they may not necessarily fully reflect the molecular mechanisms and properties of healthy human trophoblasts. Despite their disadvantage of lost proliferative capacity, we suggest that PHT cells should still be considered the first choice for placental in vitro modeling. However, a limitation of studies of this kind is that all experiments must be carried out under normoxia since lower oxygen tension drives the trophoblast differentiation along the extravillous trophoblast pathway [59]. Therefore, it should be taken into consideration that the oxygen tension may affect the basal expression and functions of transporter proteins.

## Conclusions

In conclusion, our results indicate certain regulatory mechanisms involved in monoamine transporter functionality related to trophoblast differentiation. This is likely due to phenotypic differences between the two cell stages, but the potential involvement of monoamine transporters in the process of trophoblast differentiation cannot be excluded. Importantly, our findings also provide methodological consideration for the use of in vitro models to study cellular monoamine homeostasis in the placenta.

## Supplementary Information

**Additional file 1:** **Table S1.** Clinical characteristics of pregnancies (*n* = 6) involved in the study. **Table S2.** Characterization of primary trophoblast cell purity. **Figure S1.** Raw (uncropped) representative images from Western Blot analysis. Target proteins were analysed in PHT (SERT - 1, NET - 2, DAT - 3, OCT3 - 4) and BeWo cells (SERT - 5, NET - 6, DAT - 7, OCT3 - 8).

## Data Availability

All data generated or analysed during this study are included in this published article (and its supplementary information files).

## Abbreviations

BM: Basal membrane
CTB: Cytotrophoblast
DAT: Dopamine transporter
DMEM: Dulbecco's Modified Eagle Medium
DMSO: Dimethyl sulfoxide
ELISA: Enzyme-linked immunosorbent assay
hCG: Human chorionic gonadotropin
MVM: Microvillous membrane
NET: Norepinephrine transporter
OCT3: Organic cation transporter 3
PHT: Primary trophoblast cells
SERT: Serotonin transporter
STB: Syncytiotrophoblast
