# Supplementary material for: Functional reorganization of monoamine transport systems during villous trophoblast differentiation: evidence of distinct differences between primary human trophoblasts and BeWo cells
Source: Reprod Biol Endocrinol. 2022 Aug 4;20:112. doi: 10.1186/s12958-022-00981-8 (PMC9351077; doi:10.1186/s12958-022-00981-8)
Supplement: Supplementary file 1 — Additional file 1: Table S1. Clinical characteristics of pregnancies (n = 6) involved in the study. Table S2. Characterization of primary trophoblast cell purity. Figure S1. Raw (uncropped) representative images from Western Blot analysis. Target proteins were analysed in PHT (SERT - 1, NET - 2, DAT - 3, OCT3 - 4) and BeWo cells (SERT - 5, NET - 6, DAT - 7, OCT3 - 8). [file 12958_2022_981_MOESM1_ESM.docx]

**ADDITIONAL FILES**

**Table S1. Clinical characteristics of pregnancies (n = 6) involved in the study.**

|  | Mean ± SD |
| --- | --- |
| Gestational age (weeks) | 39.31 ± 0.38 |
| Maternal age (years) | 33.60 ± 3.36 |
| Maternal BMI before pregnancy (kg/m^2^) | 24.40 ± 2.61 |
| Maternal BMI at delivery (kg/m^2^) | 31.20 ± 4.32 |
| Fetal birth weight (kg) | 3.44 ± 0.23 |
| Fetal birth height (cm) | 50.00 ± 1.41 |
| Fetal sex (M:F) | 4:2 |

**Table S2. Characterization of primary trophoblast cell purity.**

|  | Mean ± SD |
| --- | --- |
| Cytokeratin-7 | 91.80 ± 2.71 |
| Vimentin | 7.31 ± 3.37 |
| von Willebrand factor | 0.20 ± 0.28 |

*Expression levels of cytokeratin 7 (epithelial cells), vimentin (mesenchymal cells), and von Willebrand factor (endothelial cells) determined by flow cytometry (percentages of cells showing staining of indicated proteins, evaluated using FCS Express Software 7.0).*

**1 2 3**


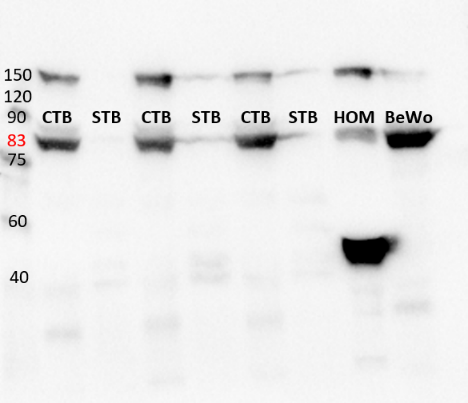

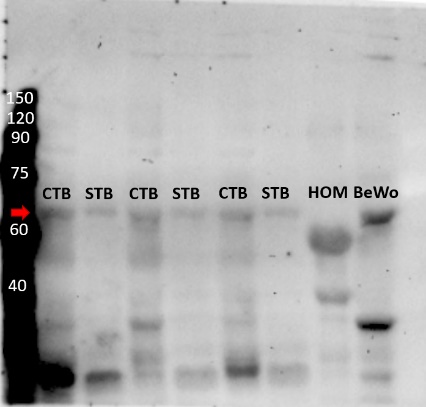

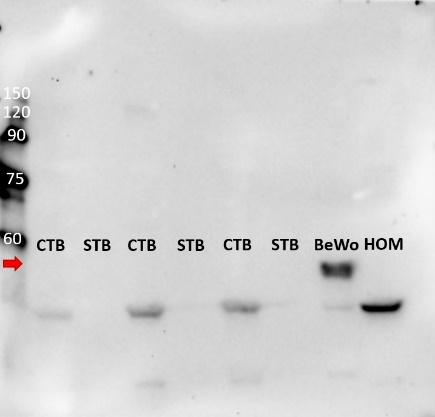


**4 5 6**


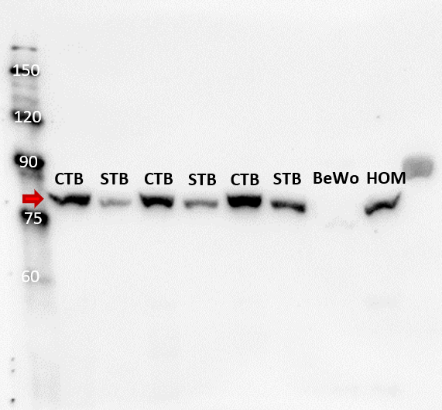

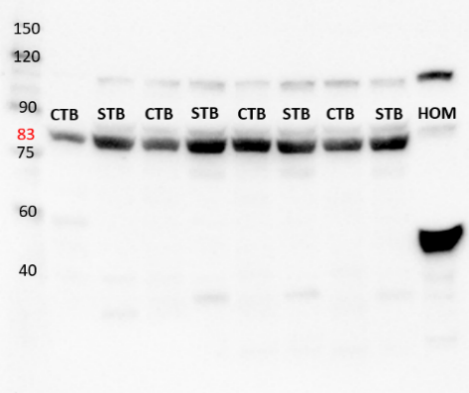

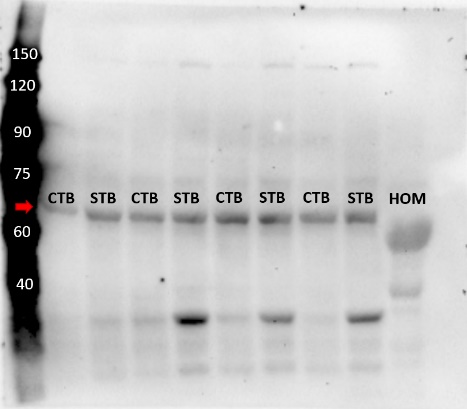


**7 8**

**
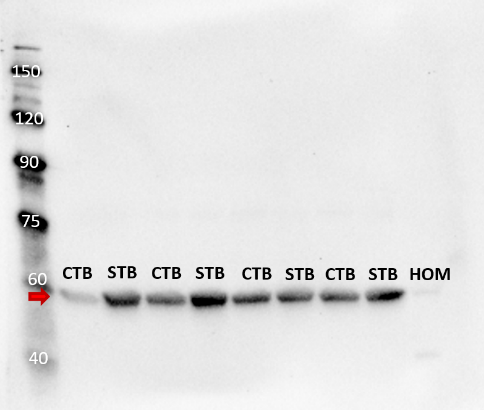

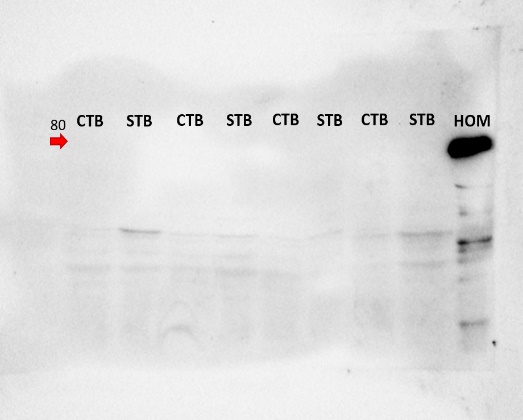
**

**Figure S1. Raw (uncropped) representative images from Western Blot analysis.** Target proteins were analysed in PHT (SERT - 1, NET - 2, DAT - 3, OCT3 - 4) and BeWo cells (SERT - 5, NET - 6, DAT - 7, OCT3 - 8).
